# Greater expression of the human leukocyte antigen-G (HLA-G) and interleukin-17 (IL-17) in cervical intraepithelial neoplasia: analytical cross-sectional study: Aumento da expressão do antígeno leucocitário humano-G (HLA-G) e interleucina-17 (IL-17) em neoplasia intraepitelial cervical: estudo transversal analítico

**DOI:** 10.1590/1516-3180.2013.7170009

**Published:** 2014-10-28

**Authors:** Lidyane Neves Miranda, Fernanda Priscila Santos Reginaldo, Daliana Maria Berenice Oliveira Souza, Christiane Pienna Soares, Tarsia Giabardo Alves Silva, Keyla Borges Ferreira Rocha, Carlos André Nunes Jatobá, Eduardo Antonio Donadi, Joanlise Marco Leon Andrade, Ana Katherine Silveira Gonçalves, Janaína Cristiana Oliveira Crispim

**Affiliations:** I BSc. Master’s Student, Department of Toxicology and Clinical Analysis, School of Pharmaceutical Sciences, Universidade Federal do Rio Grande do Norte (UFRN), Natal, Rio Grande do Norte, Brazil.; II BSc. Postgraduate Student, Department of Toxicology and Clinical Analysis, School of Pharmaceutical Sciences, Universidade Federal do Rio Grande do Norte (UFRN), Natal, Rio Grande do Norte, Brazil.; III MSc. Pharmacist, Department of Toxicology and Clinical Analysis, School of Pharmaceutical Sciences, Universidade Federal Rio Grande do Norte (UFRN), Natal, Rio Grande do Norte, Brazil.; IV PhD. Associate Professor, Department of Clinical Analysis, School of Pharmaceutical Sciences, Universidade Estadual Paulista “Júlio de Mesquita Filho” (Unesp), Araraquara, São Paulo, Brazil.; V PhD. Researcher, Department of Clinical Analysis, School of Pharmaceutical Sciences, Universidade Estadual Paulista “Júlio de Mesquita Filho” (Unesp), Araraquara, São Paulo, Brazil.; VI PhD. Assistant Professor, Department of Pathology, School of Medicine, Universidade Federal do Rio Grande do Norte (UFRN), Natal, Rio Grande do Norte, Brazil.; VII PhD. Titular Professor, Division of Clinical Immunology, Faculdade de Medicina de Ribeirão Preto (FMRP), Ribeirão Preto, São Paulo, Brazil.; VIII PhD. Associate Professor, Department of Statistics, Universidade Federal do Rio Grande do Norte (UFRN), Natal, Rio Grande do Norte, Brazil.; IX PhD. Associate Professor, Department of Obstetrics and Gynecology, School of Medicine, Universidade Federal do Rio Grande do Norte (UFRN), Natal, Rio Grande do Norte, Brazil.; X PhD, Associate Professor, Department of Toxicology and Clinical Analysis, School of Pharmaceutical Sciences, Universidade Federal do Rio Grande do Norte (UFRN), Natal, Rio Grande do Norte, Brazil.

**Keywords:** HLA-G antigens, Interleukin-17, Cervix uteri, Immunohistochemistry, Cervical intraepithelial neoplasia, Antígenos HLA-G, Interleucina-17, Colo do útero, Imunoistoquímica, Neoplasia intraepitelial cervical

## Abstract

**CONTEXT AND OBJECTIVE:**

Impaired local cell immunity seems to contribute towards the pathogenesis and progression of cervical intraepithelial neoplasia (CIN), but the underlying molecular mechanisms promoting its progression remain unclear. Identification of new molecular markers for prognosis and diagnosis of early-stage CIN may aid in decreasing the numbers of CIN cases. Several novel immunoregulatory molecules have been discovered over the past few years, including the human leukocyte antigen G (HLA-G), which through interaction with its receptors exerts important tolerogenic functions. Several lines of evidence suggest that T-helper interleukin-17 (IL-17)-producing cells (Th17 cells) may play a role in antitumor immunity. However, recent reports have implicated Th17 cells and their cytokines in both pro and anti-tumorigenic processes. The aim of the study was to evaluate the roles of HLA-G and Th17 in the immunopathogenesis of CIN I.

**DESIGN AND SETTING:**

Analytical cross-sectional study with a control group using 58 cervical specimens from the files of a public university hospital providing tertiary-level care.

**METHODS:**

We examined HLA-G and IL-17 expression in the cervical microenvironment by means of immunohistochemistry, and correlated these findings with clinical and pathological features.

**RESULTS:**

There was a greater tendency towards HLA-G and IL-17 expression in specimens that showed CIN I, thus suggesting that these molecules have a contribution towards cervical progression.

**CONCLUSION:**

These findings suggest that HLA-G and IL-17 expression may be an early marker for assessing the progression of cervical lesions.

## RESUMO

**CONTEXTO E OBJETIVO:**

A deficiência na imunidade celular localizada parece contribuir para a patogênese e progressão das neoplasias intraepiteliais cervicais (NIC), no entanto, ainda não está totalmente esclarecido o mecanismo molecular fundamental nesse processo de progressão. A identificação de novos marcadores moleculares de prognóstico e diagnóstico das NIC em estágios precoces pode ajudar a diminuir a quantidade de casos de NIC. Várias novas moléculas com função imunorregulatória foram descobertas nos últimos anos, inclusive o antígeno leucocitário humano G (HLA-G), que, através de interação com os receptores, tem importantes funções tolerogênicas. Diversas linhas de evidência sugerem que as células T-ajudantes produtoras de interleucina-17 (IL-17, células Th17), podem desempenhar um papel na imunidade antitumoral. Porém, recentes relatos implicaram as células Th17 e suas citocinas tanto em processos pro- quanto anti-tumorigênicos. O objetivo do estudo foi avaliar o papel do HLA-G e Th17 na imunopatogênese das NIC I.

**TIPO DE ESTUDO E LOCAL:**

Estudo transversal analítico com grupo controle em 58 espécimes cervicais dos arquivos de um hospital universitário público com assistência prestada no nível terciário.

**MÉTODOS:**

Avaliamos a expressão de HLA-G e IL-17 por imunoistoquímica no microambiente cervical, associando esses achados com as características clínico-patológicas.

**RESULTADOS:**

Houve tendência aumentada da expressão de HLA-G e IL-17 em espécimes que apresentaram NIC I, sugerindo que essas moléculas têm contribuição na progressão cervical.

**CONCLUSÃO:**

Estes resultados sugerem que a expressão do HLA-G e da IL-17 pode ser um marcador precoce para avaliar a progressão das lesões cervicais.

## INTRODUCTION

Impaired local cell immunity contributes towards the pathogenesis and progression of cervical intraepithelial neoplasia (CIN). The mechanism of progression from CIN to cancer has not been well explained, but intensive research has been conducted in an attempt to discover which molecules of the immune system are involved in this process, since they are known to have a very important role.^1^

Human leukocyte antigen G (HLA-G) is a non-classical class I molecule, which can be present both in membrane-bound and in soluble form, and it has been well recognized as a tolerogenic molecule, inhibiting both innate and adaptive immune responses.^2^ Under physiological conditions, HLA-G expression has limited distribution, occurring particularly in cytotrophoblast cells, where it contributes towards fetal-maternal tolerance.^3^ However, HLA-G expression may be induced under several pathological conditions, including malignant lesions, allografts and inflammatory and autoimmune disorders.^4^

Recently, studies have provided evidence that the tolerogenic protein HLA-G shows aberrant expression in a variety of cancers, and it has been suggested that this is a mechanism for tumor escape from immunosurveillance. Within the context of cervical cancer, HLA-G expression has been correlated with disease progression in patients with cervical cancer. However, the role of HLA-G in cervical premalignant and malignant lesions has not been defined clearly.^5^

Th17 cells have been characterized as interleukin (IL)-17-producing CD4(+) T cells that also produce IL-21, IL-22, and IL-26.^6^ Greater numbers of IL-17-producing cells have also been found both in peripheral blood and in tumor tissues from cancer patients at advanced stages.^7^ Although these data suggest that T-helper 17 (Th17) cells potentially have an impact on tumors, the nature and role of Th17 cells in the progression of cervical cancer remain unknown.

The presence of HLA-G in CIN patients has been correlated with a worse prognosis and less chance of survival, but the cervical expression of HLA-G and IL-17 has not been evaluated. In the present study, the possible role of HLA-G and IL-17 in the pathogenesis and progression of cervical lesions was investigated. We measured HLA-G and IL-17 expression and correlated their levels in CIN I and chronic cervicitis (CC) patients with the clinical and pathological features, by means of immunohistochemistry. This study may help in understanding the possible roles of coexpression of HLA-G and IL-17 in the progression of cervical lesions.

## OBJECTIVES

The aim of this study was to assess the expression of human leukocyte antigen-G and interleukin-17 in cervical intraepithelial neoplasia.

## METHODS

### Patients

The study protocol was approved under No. 526/11 by the Ethics Committee of Hospital Universitário Onofre Lopes (HUOL), Universidade Federal do Rio Grande do Norte (UFRN). Cervical biopsies obtained from 58 patients were selected from the archives of the Pathology Department, UFRN School of Medicine, Brazil, during the years 2006-2009. Out of these patients, 35 were confirmed as presenting CIN I and 23 had CC. Clinical and pathological information about the patients, such as age, histories of smoking and alcohol consumption, contraceptive method, education level, age at first intercourse, ethnicity, number of sexual partners during lifetime, parity and number of abortions, was obtained from the patients’ medical records.

### Histology

Cervical biopsies are routinely performed in our gynecology unit. Fifty-eight cervical specimens were obtained. All biopsy material was prepared using hematoxylin and eosin (HE) staining for analysis and was classified by a single pathologist as prescribed by Richart for diagnosing CIN.^8^

The CIN terminology divides cervical cancer precursors into three groups: CIN I corresponds to lesions previously diagnosed as mild dysplasia; CIN II corresponds to moderate dysplasia; and CIN III to both severe dysplasia and carcinoma *in situ*, since pathologists could not reproducibly distinguish between the two. At the time of introducing the CIN system, it was thought to define a spectrum of histological abnormalities that shared common etiology, biology and natural history.^9^ In cases of chronic cervicitis, round cells (including lymphocytes, plasma cells and histiocytes) predominate in the inflammatory infiltrate and are associated with varying amounts of granulation tissue and stromal fibrosis. The diagnosis of chronic cervicitis should be reserved for cases in which there is definite clinical and histological evidence of a significant chronic inflammatory process.^10^

The 20 cervical biopsies from healthy women were kindly provided by the gynecology department of Faculdade de Medicina de Ribeirão Preto (FMRP).

### Immunohistochemistry

Sections of thickness 3 µm were cut, placed on organosilane-pretreated slides, and subjected to immunohistochemical assay using two monoclonal antibodies: 5A6G7 (EXBIO, Vestec, Czech Republic) against soluble HLA-G5, diluted at 1:50; and ebio65DEC17 (Ebioscience, San Diego, California, USA), which reacts with human IL-17A, diluted at 1:100. The cervical specimens were dewaxed in xylene, rehydrated in a graded alcohol series and rinsed in water. For antigen retrieval, the sections were immersed in 10 mM sodium citrate buffer (pH 6.2). Endogenous peroxidase blocking was performed using 3% hydrogen peroxide. Nonspecific binding was performed using 3% low-fat dried milk diluted 1:100 in phosphate-buffered saline (PBS). The slides were incubated with the primary monoclonal antibody in a humidified chamber at 4 ºC overnight. Next, MACH 4 Universal HRP Polymer Detection (Biocare Medical, California, USA) was added and incubated for 30 minutes. Finally, the samples were incubated with 3,3-diaminobenzidine (DAB, Gibco; Gaithersburg, Maryland, USA), lightly counterstained with Harris hematoxylin for 15 seconds, exhaustively rewashed with tap water, air-dried and mounted using Permount mounting medium (Merck, Darmstadt, Germany).

To validate the anti-HLA-G monoclonal antibody (mAb) and the immunohistochemical method, we systematically analyzed paraffin-embedded sections of trophoblastic tissue (positive control). To validate the anti-IL-17, we systematically analyzed paraffin-embedded sections of laryngeal tissue (positive control). The baseline expression of these molecules was evaluated by means of twenty cervical biopsies on healthy patients without cytological abnormalities, which were obtained from the Department of Gynecology, FMRP. The negative control was prepared by omitting the primary antibody.

The immunohistochemical evaluation of protein expression was done through analysis by a pathologist. The findings were classified according to the quantity of labeled cells and the intensity of their expression pattern. An average of 10 fields at 400 x magnification was used for microscopic evaluation of immunostaining, on each histological section. For quantification, the expression of the markers used was scored as follows: 0 for no expression; 1 for 1-30% positive cells; 2 for 31-70% positive cells; and 3 for 71-100% positive cells. This classification system was based on earlier work by Xie et al.^11^

### Statistical analysis

The chi-square test or Fisher’s exact test was used to test for associations between: 1) qualitative clinical or demographic variables (education level, ethnicity, smoking habit, alcohol use and contraceptive method used) and expression of HLA-G (present or absent) and IL-17 (present or absent); and 2) histopathological findings and expression levels (Tables 1 and 2) relating to HLA-G (0%, 1-30%, 31-70% and 71-100%) and IL-17 (0%,1-30%, 31-70% and 71-100%).

**Table 1.**
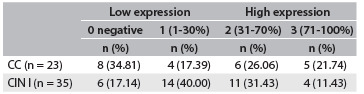
Quantitative distribution of expression of human leukocyte antigen G (HLA-G) in cervical precursor lesions

Chi-square test for independent samples, P = 0.1604. CC = chronic cervicitis; CIN = cervical intraepithelial neoplasia.

**Table 2.**
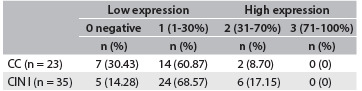
Quantitative distribution of expression of IL-17 in cervical precursor lesions

Chi-square test for independent samples, P = 0.2735. CC = chronic cervicitis; CIN = cervical intraepithelial neoplasia.

The means of the quantitative clinical or demographic variables (age, age at first intercourse, number of sexual partners during lifetime, parity and number of abortions) were compared between the CC and CIN I groups by means of two-sample t tests.

Logistic regression analysis was performed to assess the effect of HLA-G and IL-17 expression (present or absent) on the odds of presenting CIN I (in comparison with CC), after adjustment for relevant clinical and demographic variables. These included age, age at first intercourse, number of sexual partners during lifetime, parity and oral contraceptive used.

The statistical analyses were conducted using the GraphPad InStat software (San Diego, California, USA) or R version 2.12.2. Tests yielding P-values < 0.05 were considered significant.

## RESULTS

### Characteristics of the study population

The clinical and pathological variables of the participants studied are shown according to histological group in Table 3. The average ages of the CC and CIN I patients were 38.21 years (standard deviation, SD = 14.43) and 33.02 years (SD = 10.61), respectively. In both groups, the majority of the participants were non-Caucasian and had completed high school. The CC and CIN I patients were comparable regarding their age at first sexual intercourse and the number of sexual partners during their lifetimes, but CIN I cases were more likely to have had a higher number of sexual partners during their lifetimes. In relation to cigarette smoking and alcohol use, both groups showed a few users. CC patients were likely to be regular users of contraceptive methods.

**Table 3.**
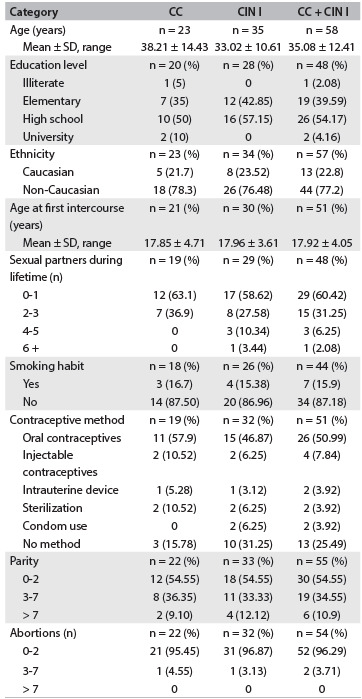
Clinical and pathological variables observed in patients with cervical lesions

CC = chronic cervicitis; CIN = cervical intraepithelial neoplasia; SD = standard deviation.

### Histology

In the present study, we used immunohistochemical staining to analyze the expression and cell location of HLA-G and IL-17 in 58 cervical specimens. From the histopathological findings, the cervical tissue samples were stratified as CIN I patients (n = 35) or CC patients (n = 23).

### Expression of HLA-G and IL-17 in cervical precursor lesions

**Figure 1.**
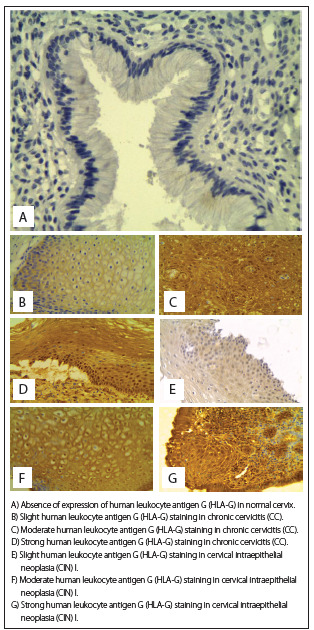
Human leukocyte antigen G (HLA-G) expression in cervical epithelium was analyzed by means of immunohistochemistry. Labeling was accomplished using anti-HLA-G 5A6G7 mAb (EXBIO, Czech Republic).

**Figure 2.**
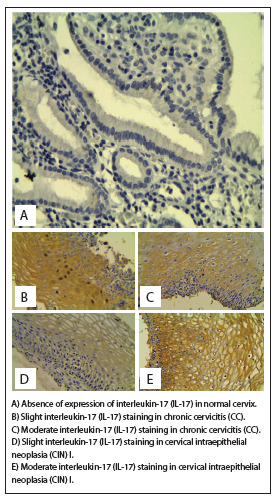
Interleukin-17 (IL-17) expression in the cervical epithelium was analyzed by means of immunohistochemistry. Labeling was accomplished using anti-IL-17 (Ebioscience, San Diego, California, USA).

To explore whether HLA-G and IL-17 might be involved in the CIN cases, we first examined whether HLA-G and IL-17 were present in cervical specimens. In the whole group, HLA-G molecules were detected in 44 cases (75.86%). Among specimens that presented HLA-G expression, 29 out of 35 (82.86%) exhibited CIN I, and 15 out of 23 (65.22%) exhibited CC. Similarly, IL-17 molecules were detected in 46 cases (79.31%). Considering only the patients who presented IL-17 expression, 30 out of 35 patients (85.71%) exhibited CIN I and 16 out of 23 (69.56%) exhibited CC. Absence of HLA-G and IL-17 expression was observed in the control group, as shown in Figures 1 and 2, and in Table 4.

**Table 4.**
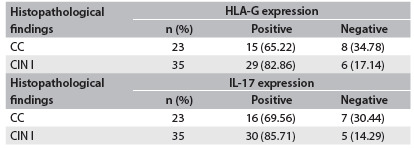
Association between human leukocyte antigen G (HLA-G) and interleukin-17 (IL-17) status and histopathological findings

CC = chronic cervicitis; CIN = cervical intraepithelial neoplasia.

After adjustment for other covariables, the occurrences of HLA-G were significant (P-value = 0.04), with odds ratio (OR) estimated as 6.61 (95% confidence interval, CI: 1.22-49.55). This indicated that the odds of having CIN I (compared with CC) was 6.61 times greater (or 661% greater) for women who expressed HLA-G than for those who did not. Age was the only other significant predictor, after adjustment for other covariables, with an OR of 0.87 (95% CI: 0.75- 0.96, P-value = 0.02), thus indicating a protective effect. In other words, the older the patient was, the smaller the odds of having CIN I also were. None of the other covariables were significant (Table 5).

**Table 5.**
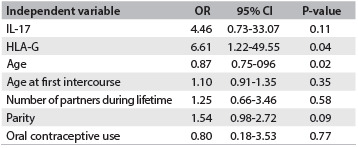
Logistic regression analyses on cervical intraepithelial neoplasia (CIN) I status. Model adjusted according to interleukin-17 (IL-17), human leukocyte antigen G (HLA-G), age, age at first intercourse, number of partners during lifetime, parity and oral contraceptive use

OR = odds ratio; CI = confidence interval.

HLA-G expression was primarily detected in the epithelial cells, fibroblasts and lymphocytes, and a standard dial-type cytoplasmic membrane was maintained. HLA-G was strongly expressed in trophoblastic slices that were used as positive controls, while HLA-G expression was not found in any specimens obtained from healthy controls.

## DISCUSSION

Cervical squamous intraepithelial lesions are a precancerous stage of cervical cancer.^12^ The mechanism that promotes the progression of cervical lesions has not been clearly explained, but the immune response appears to be an important factor.^5^ Therefore, identification of new molecular markers to improve clinical diagnosing of early-stage cervical lesions is still necessary and may enable more effective evaluation of patients with early-stage lesions, thereby resulting in slower progression of these lesions.

In the cervical context, Guimarães et al. reported that HLA-G expression was low in cervical cancer specimens.^13^ On the other hand, Zheng et al. reported that HLA-G is abundantly expressed in premalignant and malignant cervical intraepithelial lesions.^5^

Thus, our study found that HLA-G expression was a significant predictor in relation to CIN I and age, such that overall, HLA-G was present in approximately 75.86% of the cases and was primarily detected in epithelial cells, fibroblasts and lymphocytes. The presence of HLA-G was not influenced by other factors, such as sex, ethnicity, number of partners during lifetime, parity and oral contraceptive use. This suggests that the HLA-G molecule could be associated with the progression of cervical lesions.

In addition, polymorphic sites of HLA-G genes in cervical lesions and cancer have been studied. In high-grade and invasive cervicovaginal cancer patients, the 14 base pair (bp) In/In polymorphism seems to be associated with greater development of invasive cervical cancer.^14^ On the other hand, spontaneous demethylation events in the HLA-G promoter do not play a primary role in promoting escape from immunosurveillance, in relation to development of precancerous cervical lesions.^15^

To our knowledge, the present study is the first to explore the HLA-G and IL-17 expression profile in the cervical microenvironment in CIN I patients, in whom more than half of the biopsies (75.86%) exhibited HLA-G expression. The presence of HLA-G, which was significantly associated with CIN and age, was not influenced by other variables. Thus, the data shown here certainly contribute towards shedding some light on the physiopathology of CIN, in which HLA-G expression in cervical cells may act in conjunction with other factors, such as immunosuppression induced by HPV infection, thereby resulting in the more severe cervical disease observed in CIN III and cervical cancer patients.

Since the first description of HLA-G expression, its association with malignant lesions has been intensively studied. The data available so far have shown that the vast majority of tumors may express varying degrees of HLA-G isoforms, thus reflecting a potential immune escape mechanism. It is also worth mentioning that HLA-G expression is highly dependent on tumor microenvironment factors, particular when IL-10 and a hypoxic factor are present.^16^

The role of IL-17 in the tumor microenvironment is still controversial. Information about the behavior of cytokines in CIN cases is scarce. Previous studies have shown that patients with uterine cervical cancer (UCC) have a higher proportion of Th17 cells. Notably, in UCC patients, increased Th17 prevalence has been correlated with clinical stage, lymph node metastases and vasoinvasion.^7^

Our study found that IL-17 expression was greater in CIN I cases. IL-17 molecules were detected in 46 cases (79.31%) and, when considering only the patients that presented IL-17 expression, 30 out of 35 (85.71%). Recently, in patients with ovarian cancer, Lan et al. showed that the IL-17 levels were significantly greater in ovarian cancer cases than in normal ovarian tissues (P < 0.001).^17^ Moreover, ovarian tumor antigen-specific CD4(+) T cells secrete high levels of IL-17.^18^ However, the exact role of IL-17 in tumor immunopathogenesis remains undefined. It has been reported that expression of interleukin-17 in tumor cells suppresses tumor progression through enhanced antitumor immunity or promotes tumor progression through an increase in inflammatory angiogenesis.^19^

Many studies have discussed the role of HLA-G and IL-17 in various types of cancer.^16^^,^^20^ However, this is the first study to correlate the expression of two molecules that are important in the early stages of the cervical lesion. Consistently, our study demonstrated that coexpression of HLA-G and IL-17 was implicated in the pathogenesis and progression of cervical lesions (CC and CIN I), correlating these findings with clinical and pathological features.

## CONCLUSION

Taken together, our data suggest that in CIN I patients, the increased HLA-G levels could be correlated with progression of cervical lesions, and that presence of IL-17 may be a useful indicator for representing the severity of tissue injury. Thus, the data suggest that Th17 cells are mediators during the immunological process of CIN development, thereby indicating that HLA-G and IL-17 potentially have a role in the development and progression of cervical lesions.

## References

[1] Burd EM (2003). Human papillomavirus and cervical cancer. Clin Microbiol Rev.

[2] Carosella ED, Favier B, Rouas-Freiss N, Moreau P, Lemaoult J (2008). Beyond the increasing complexity of the immunomodulatory HLA-G molecule. Blood.

[3] Rouas-Freiss N, Gonçalves RM, Menier C, Dausset J, Carosella ED (1997). Direct evidence to support the role of HLA-G in protecting the fetus from maternal uterine natural killer cytolysis. Proc Natl Acad Sci U S A.

[4] Carosella ED, Moreau P, Lemaoult J, Rouas-Freiss N (2008). HLA-G: from biology to clinical benefits. Trends Immunol.

[5] Zheng N, Wang CX, Zhang X (2011). Up-regulation of HLA-G expression in cervical premalignant and malignant lesions. Tissue Antigens.

[6] Bettelli E, Carrier Y, Gao W (2006). Reciprocal developmental pathways for the generation of pathogenic effector TH17 and regulatory T cells. Nature.

[7] Zhang Y, Ma D, Zhang Y (2011). The imbalance of Th17/Treg in patients with uterine cervical cancer. Clin Chim Acta.

[8] Richart RM (1986). The incidence of cervical and vaginal dysplasia after exposure to DES. JAMA.

[9] Wright TC, Ronnett BM, Kurman RJ, Ferenczy A, Kurman RJ, Ellenson LH, Ronnett BM (2011). Precancerous lesions of the cervix. Blaustein’s pathology of the female genital tract.

[10] Wright TC, Ronnett BM, Ferenczy A, Kurman RJ, Ellenson LH, Ronnett BM (2011). Benign diseases of the cervix. Blaustein’s pathology of the female genital tract.

[11] Xie X, Clausen OP, Boysen M (2004). Bag-1 expression as a prognostic factor in tongue squamous cell carcinomas. Laryngoscope.

[12] Lukovic L, Milasin J (1992). Sister chromatid exchanges in patients with carcinoma in situ of cervix uteri. Cancer Genet Cytogenet.

[13] Guimarães MC, Soares CP, Donadi EA (2010). Low expression of human histocompatibility soluble leukocyte antigen-G (HLA-G5) in invasive cervical cancer with and without metastasis, associated with papilloma virus (HPV). J Histochem Cytochem.

[14] Ferguson R, Ramanakumar AV, Koushik A (2012). Human leukocyte antigen G polymorphism is associated with an increased risk of invasive cancer of the uterine cervix. Int J Cancer.

[15] Gillio-Tos A, Bicalho Mda G, Fiano V (2012). Case-control study of HLA-G promoter methylation status, HPV infection and cervical neoplasia in Curitiba, Brazil: a pilot analysis. BMC Cancer.

[16] Singer G, Rebmann V, Chen YC (2003). HLA-G is a potential tumor marker in malignant ascites. Clin Cancer Res.

[17] Lan C, Huang X, Lin S (2013). High density of IL-17-producing cells is associated with improved prognosis for advanced epithelial ovarian cancer. Cell Tissue Res.

[18] Cannon MJ, Goyne HE, Stone PJ (2013). Modulation of p38 MAPK signaling enhances dendritic cell activation of human CD4+ Th17 responses to ovarian tumor antigen. Cancer Immunol Immunother.

[19] Kryczek I, Banerjee M, Cheng P (2009). Phenotype, distribution, generation, and functional and clinical relevance of Th17 cells in the human tumor environments. Blood.

[20] Wägsäter D, Löfgren S, Hugander A, Dimberg J (2006). Expression of interleukin-17 in human colorectal cancer. Anticancer Res.

